# Early life differences in behavioral predispositions in two Alligatoridae species

**DOI:** 10.1007/s10071-020-01461-5

**Published:** 2021-01-17

**Authors:** Stephan A. Reber, Jinook Oh, Judith Janisch, Colin Stevenson, Shaun Foggett, Anna Wilkinson

**Affiliations:** 1 School of Life Sciences, University of Lincoln, Lincoln, UK; 2 Department of Cognitive Biology, University of Vienna, Vienna, Austria; 3 Present Address: Lund University Cognitive Science, Lund University, Lund, Sweden; 4 Institute of Science and Technology Austria, Klosterneuburg, Austria; 5 Department of Interdisciplinary Life Sciences, University of Veterinary Medicine Vienna, Vienna, Austria; 6 Crocodiles of the World, Brize Norton, UK

**Keywords:** Behavioral predisposition, *Caiman crocodilus*, *Alligator mississippiensis*, Crocodilian, Exploration, Neophobia

## Abstract

Behavioral predispositions are innate tendencies of animals to behave in a given way without the input of learning. They increase survival chances and, due to environmental and ecological challenges, may vary substantially even between closely related taxa. These differences are likely to be especially pronounced in long-lived species like crocodilians. This order is particularly relevant for comparative cognition due to its phylogenetic proximity to birds. Here we compared early life behavioral predispositions in two Alligatoridae species. We exposed American alligator and spectacled caiman hatchlings to three different novel situations: a novel object, a novel environment that was open and a novel environment with a shelter. This was then repeated a week later. During exposure to the novel environments, alligators moved around more and explored a larger range of the arena than the caimans. When exposed to the novel object, the alligators reduced the mean distance to the novel object in the second phase, while the caimans further increased it, indicating diametrically opposite ontogenetic development in behavioral predispositions. Although all crocodilian hatchlings face comparable challenges, e.g., high predation pressure, the effectiveness of parental protection might explain the observed pattern. American alligators are apex predators capable of protecting their offspring against most dangers, whereas adult spectacled caimans are frequently predated themselves. Their distancing behavior might be related to increased predator avoidance and also explain the success of invasive spectacled caimans in the natural habitats of other crocodilians.

**Supplementary Information:**

The online version contains supplementary material available at 10.1007/s10071-020-01461-5.

## Introduction

The comparative approach is one of the main methods used to study the evolution of cognition (Tinbergen 1963). Cognitive capacities can be traced through time and their origins better understood by examining similarities and differences between different species in various positions in the tree of life. However, closely related species may also differ greatly in their cognition. Factors that could be involved in producing such differences are the behavioral predispositions of the species. A species that is more likely to explore novel stimuli in its surroundings may learn more rapidly than one that is less likely to do so. Behavioral predispositions may vary due to ecological differences rather than phylogenetic distance, resulting in quite different cognitive abilities being observed in closely related species. It is, therefore, important to consider such factors to draw lasting conclusions from comparisons of cognition across different taxa (MacLean et al. 2012).

Behavioral predispositions, e.g., an innate tendency to freeze when facing a potentially dangerous situation, may markedly increase an organism’s chances of survival (Gray 1987; Vilhunen and Hirvonen 2003). This could be particularly relevant very early in life, as behavioral predispositions may decrease the risk of predation before an animal has had time to learn about the threats in its environment (Tierney 1986; Hawkins et al. 2004). Such innate behavioral traits may subsequently be shaped by life experience, the extent to which this occurs depending on the animal’s behavioral plasticity (Gumbert 2000; Kelley and Magurran 2003). For instance, neonate cottonmouths (*Agkistrodon piscivorus*) do not habituate to a non-harmful predatory stimulus while adults, exposed to the same stimulus, show a reduction in their tendency to strike over time (Glaudas et al. 2006). Behavioral dispositions can also differ between animals of the same taxonomic order (Fraser and Gilliam 1987), populations of the same species (Wilson et al. 1993; Bell and Stamps 2004) and even individuals from the same clutch when exposed to different conditions in ovo (Rokka et al. 2014; Siviter et al. 2017). These differences are likely to reflect survival strategies dictated by specific challenges in the environments they inhabit (Greenberg and Mettke-Hofmann 2001). Furthermore, behavioral predispositions can also change drastically across an animal’s life span (Kendal et al. 2005; Miller et al. 2015). It is, therefore, likely that such differences would be most pronounced in long-lived species that exhibit significant ontogenetic changes in their feeding and social ecology. A great example are crocodilians, where some species increase their body size by 3–5 orders of magnitude (Radloff et al. 2012), preferential prey species can shift from insects to large ungulates throughout life (Cott 1961), and juveniles seek safety in numbers, while adults of several species are highly territorial (Grigg and Kirshner 2015).

Crocodilians are the closest living relatives of birds and both groups share a common ancestor with all extinct dinosaurs (Hugall et al. 2007). Their brain structure is highly similar to birds but physiologically they resemble other non-avian reptiles and mammals (Grigg and Kirshner 2015). This makes them an interesting order for understanding the evolutionary origin of avian cognition in particular (Vergne et al. 2009; Reber et al. 2017) and for the comparative approach in general. Crocodilians are widespread across the globe but have relatively few surviving species (currently 28 are recognized; Stevenson 2019; Murray et al. 2019). They share a highly conserved body plan, a semi-aquatic life history, and a seemingly identical call repertoire (Webb et al. 1987; Britton 2001; Reber 2018). It is, therefore, tempting to assume that crocodilian species do not differ greatly in behavior. However, they face different challenges in their respective environments. This can depend on the prey they hunt, the predators they are exposed to, and the seasonal changes they have to cope with, e.g., the avoidance of drought in Nile crocodiles (*Crocodylus niloticus*; Kofron 1993); or the risks of hibernation in Chinese alligators (*Alligator sinensis*; Thorbjarnarson and Wang 2010). Thus, crocodilians are likely to differ in their overall behavioral predispositions. Observations in the wild and in captivity have revealed that different species behave differently towards conspecifics and other entities in their environment (Garrick and Lang 1977; Trutnau and Sommerland 2006). There are, however, only few experimental comparisons. For instance, in a serial reversal learning study, American crocodiles (*Crocodylus acutus*) produced significantly fewer errors than American alligators (*Alligator mississippiensis*) for each reversal (Gossette and Hombach 1969). But the latter species showed consistently shorter latencies to make a choice in a trial. The authors suggested that the alligators were more motivated to participate than the crocodiles, which might have led to more errors. This difference is particularly interesting, because the two species overlap in their geographical range and have no natural predators as adults; they do, however, occupy different ecological niches. American crocodiles are commonly found in coastal areas and frequently hunt in marine habitats, whereas American alligators predominantly inhabit inland habitats and rarely swim in saltwater (Stevenson 2019). It is, therefore, conceivable that their differing performances in cognitive tasks could be explained by the differences in behavioral ecology between the two species.

All crocodilians are highly susceptible to predation in the first months of life by a large variety of other animals, including large fish, snakes, monitor lizards, raptors, wading birds, small mammalian carnivores, and also conspecifics (Somaweera et al. 2013). However, members of certain species (e.g., saltwater crocodile *Corocodylus porosus*, American alligator) become apex predators in their respective habitats when they reach maturity (Grigg and Kirshner 2015), while others (e.g., Yacare caiman *Caiman yacare*) remain susceptible to predation into adulthood (Azevedo and Verdade 2012). Because of the similar risks of predation in early life, one might predict that hatchlings of any species would show similar responses to novel stimuli, such as little exploration behavior in a novel environment and overall lower levels of activity. After reaching a less vulnerable body size, crocodilians have a far smaller range of potential predators (Somaweera et al. 2013) and could be expected to display higher levels of activity and increased exploration behavior. Although this has, to our knowledge, not yet been studied in crocodilians, similar dispositions have been described in their closest living relatives, birds: species facing higher predation pressure are less explorative and more neophobic (Heinrich 1995; Greenberg and Mettke-Hofmann 2001), and growing evidence suggests that these traits might vary more between age-classes than between species (O’Hara et al. 2017).

Some crocodilian species are critically endangered and reintroduction is either recommended or ongoing (Wang et al. 2011; Kanwatanakid-Savini et al. 2012). To increase the potential success of such conservation efforts, it is vital to determine whether crocodilians adapt their behavior in a developmentally dependent manner. For instance, if a species shows high levels of exploratory behaviors early in life despite still being vulnerable to a large spectrum of predators, it would be advisable to raise the juveniles to a larger body size before their release, whereas that might not be necessary for members of a species with stronger predispositions for anti-predator behaviors. Conversely, some crocodilians have become successful invasive species in other crocodilians’ natural habitat, negatively affecting local populations (Ellis 1980). In such cases, population management efforts could benefit from a better understanding of early life behavioral predispositions and whether hatchlings of an invasive species might have an advantage due to stronger intrinsic predator avoidance or superior competitive abilities (Hudina et al. 2015).

We investigated behavioral predispositions of American alligator and spectacled caiman (*Caiman crocodilus*) hatchlings using well-established experimental methods (Réale et al. 2007). We aimed to determine whether i) individuals from these two species display consistent behavioral traits at a very young age and ii) whether there were differences between the two species. All subjects in the present study were the same age and maintained under the same conditions prior to and during the experiments. In an initial phase (Phase 1) all animals were exposed to three conditions: novel object, novel environment: open field, and novel environment: shelter. The proximity to a novel object in a familiar environment, but in the absence of an additional positive stimulus (e.g., food), can be used as a measure for exploration behavior (Greggor et al. 2015). The range of movement in a novel environment serves as an assessment of activity. Shelter usage served as a control, i.e., to determine whether high levels of movement in the novel environment: open field trials were actually indicators of activity levels in unfamiliar surroundings, and thereby possible exploration behavior, or whether animals primarily wanted to escape open space. All conditions were then repeated a week later (Phase 2) to investigate whether individual hatchlings showed behavioral consistency over time.

While adult American alligators have no natural enemies, adult spectacled caimans have a number of predators, such as jaguar, cougar, and green anaconda (Calle et al. 1994; Scognamillo et al. 2003). Should juveniles of the two species already exhibit behavioral predispositions similar to those of adults, we could expect them to show differential behaviors in our conditions; e.g., alligators might be more explorative. In addition, spectacled caimans are a successful invasive species in many areas, including the Everglades, a natural habitat of the much larger American alligator (King and Krakauer 1966). In regions where these two species cohabitate, spectacled caiman juveniles are hence confronted with an additional predator against which a guarding parent cannot provide effective protection. Predispositions for increased anti-predator behaviors, e.g., reduced activity in novel environments, could consequently increase the caimans’ survival chances. Therefore, we could expect to observe differences in behavioral predispositions in young members of these two Alligatoridae species.

## Methods

### Subjects

The experimental subjects were 11 American alligator and 11 spectacled caiman hatchlings. The animals were too young to identify their sex. As crocodilians have temperature-dependent sex determination (Grigg and Kirshner 2015), the presumed sex of the subjects is based on their incubation temperature. The alligators hatched on the 8th September (5 individuals, 70 days of incubation, average incubation temperature = 32.42 °C, presumed males) and 16th September (6 individuals, 79 days of incubation, average incubation temperature = 29.8 °C, presumed females). The caimans all hatched on the 17th September (11 individuals, 75 days of incubation, average incubation temperature = 31.8 °C, presumed males; Ferguson and Joanen 1982). All subjects were left in the incubator for 2 days to fully absorb the remaining yolk. Each incubation group was then transferred into a transparent plastic enclosure (48 × 39 × 31 cm), filled with water which was changed daily to let the naval openings seal under hygienic conditions. Afterwards, the hatchlings were kept in glass vivaria with 5-cm high depth water, a heat-lamp, and a brick as a dry basking spot. All subjects could be individually recognized by their distinctive hide markings. The animals were well habituated to human handling. At the start of the experiment the alligators were between 26 and 32 days, and the caimans between 27 and 28 days old.

### Experimental setup

Two plastic arenas (70 × 55 × 37 cm) with lids were used to run the experiment. Five optically different environments were created; two from the arena’s original colors (black, blue) and three in which the walls of the boxes were covered with colored wrapping paper (cyan with white dots, rose with flower pattern, white with silver stars). The floor of each arena was fully lined with corrugated cardboard to reduce light reflection. Each arena was covered with a lid upon which an LED bulb was attached (light bulb: Philips Master LEDbulb 7 W, 470 lm). The animals’ behavior was recorded using a GoPro (Hero4 silver edition, https://gopro.com, 60 frames/s, image size: 1920 × 1080) through a small hole in the lid of the arena.

### Experimental procedure

#### Novel object

Two days before the start of the experiment, subjects were habituated to the novel object arena (one of the two boxes without wrapping paper on the walls, environments counterbalanced across animals). On the first day, they were allowed to explore the box with other animals (2–4 conspecifics in the arena simultaneously) for 20 min. Crickets and mealworms were offered in the arena (8 alligators and 8 caimans showed hunting behavior). On the second day, each subject spent 20 min alone in the arena. Again, food was offered. If the subject did not display hunting behavior (chasing after or jumping towards food) in the first 20 min, they were given a break and later on placed into the arena once more for 20 min (7 alligators, 10 caimans) to ensure they were habituated to the environment.

**Fig. 1:**
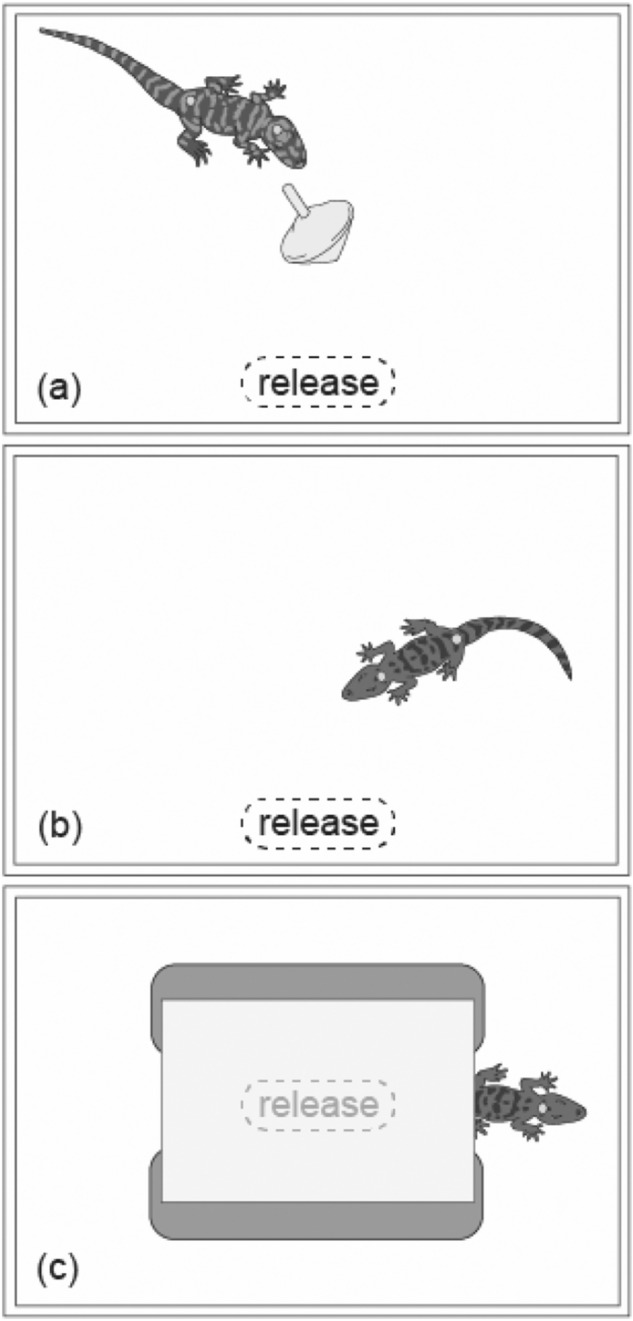
Three conditions (not to scale). An American alligator during a novel object trial (**a**). A spectacled caiman in a novel environment: open field trial (**b**). A spectacled caiman in a novel environment: shelter trial (**c**). For each condition the release location is indicated

During a novel object trial a small object was put in the middle of the arena, either a blue toy car or a yellow spinning top (see Table S1, Online Resource 1 for details). The specific object presented was counterbalanced across subjects and phases (subjects saw a different object in each phase). At the onset of a trial the hatchling was placed in a starting area close to the center of one of the longer sidewalls (Fig. 1a).

#### Novel environment: open field

This was identical to the previous condition but with some key changes. Instead of novel objects, different novel environments were used. The environments were created by changing the walls of the arena which could either be plain or covered with wrapping paper. This ensured an unfamiliar environment for the subject. At the start of each trial a hatchling was placed in a starting area close to the center of one of the longer sidewalls (Fig. 1b).

#### Novel environment: shelter

This was identical to the novel environment: open field condition except that animals had access to a shelter. The shelter was a white tile laid onto two small bricks and was positioned at the center of the arena. At the onset of the trial a hatchling was placed under the shelter (Fig. 1c).

#### Testing schedule across two phases

All trials took place either in the morning or the evening hours; this was consistent within a phase but counterbalanced across phases for each individual. Each phase contained three trials (one for each condition) with one 10-min trial being run per day. On each day, an animal received a trial of a different condition, with order of conditions counterbalanced across individuals. The second phase commenced a week after the first and the order of conditions was the same for a given individual in both phases. The different novel objects and environments were counterbalanced across individuals for the two phases. For example, spectacled caiman #3 was always tested in the morning hours in Phase 1. On the first day, it participated in a ‘Novel Environment: Open Field’ trial, on the second in a ‘Novel Environment: Shelter’ trial, and in a ‘Novel Object’ trial on the third. After 4 days without a trial, Phase 2 began. Now caiman #3 was always tested in the evening hours. It again participated in one trial per day with the same order of the conditions as in the first phase. However, the walls of the arena in the ‘Novel Environment: Open Field’ and ‘Novel Environment: Shelter’ trials looked different than in the previous phase; and the novel object in the ‘Novel Object’ trial was changed as well (see Table S1, Online Resource 1 for details).

Before each trial, the floor of the arena was wiped with a damp cloth, the subject was removed from its home vivarium, dried off using paper towels, and two small round adhesive stickers (1 cm diameter) were placed on its head (red or blue) and tail-base (green) to facilitate automated video analysis. All trials were recorded and video recording was started immediately prior to the animal being introduced into the arena. After the 10-min trial time, recording was stopped, the subject was removed from the arena, the stickers carefully removed and then it was returned to its home vivarium.

### Automated video analysis

Each frame (60 f/s) was first exported as an image (jpg). A custom color tracking software (“AMA”, Alligatoridae Motion Analyzer, available online: https://github.com/jinook0707/AMA), used the color stickers on the head and tail-base to identify the positions of these body-parts and recorded their coordinates in number of pixels (*x*-, *y*-axis of the entire video frame). If the animal was fully or partially in the shelter, one or both color tags could not be detected. A pixel edge length equaled roughly 1.278 mm for the chosen resolution (image size: 960 × 540, reduced from original video to increase processing speed) and the distance from the arena floor was 37 cm. Both were kept constant across trials. Additionally, the distance (again in pixels) between the head tag and the center of the arena (location of the object in the novel object condition) was noted.

To obtain movement data, the software compared each individual frame (*f*_*i*_) with a frame (*f*_*i−30*_) from half a second ago (30 frames). Only if the virtual “head line” (HL) connecting the head tag’s (*ht*_*i*_) position in the current *f*_*i*_ and the head tag’s (*ht*_*i−30*_) position from the previous *f*_*i−30*_ had changed in length by a minimum of 5 pixels (~ 6.39 mm), then the software recorded the distance of this new position from the previously recorded position. Because of minute movements below the threshold and small changes in the distance between the camera lens and the subject, this recorded distance was usually larger than 5 pixels. The software also automatically recorded these movement behaviors as ‘walking distance’ or as ‘head movements without walking’. To determine which of the two behaviors had occurred, the program looked at the virtual “tail-base line” (TbL) that, equivalent to HL, connects the position of the tail-base tags (*tbt*_*i*_ and *tbt*_*i−30*_) of the two frames (*f*_*i*_ and *f*_*i−30*_). The angles of HL (AHL) and TbL (ATbL) relative to the whole frame were calculated (e.g., straight to the right = 0°, straight up = 90°, straight down = − 90°). If the absolute difference between the two angles (AHL-ATbL) was smaller than 45° (a), the pixel difference was counted as “walking distance”; if the difference exceeded the 45°-threshold (b), the pixel margin was recorded as “head movements without walking” (see Fig. S2, Online Resource 1). These two measures were mutually exclusive, because crocodilians have to keep their head stable during locomotion on land; a head turn can only be performed from a stable position.

### Visual coding check

To check the accuracy of the data, a researcher (JJ) screened the automated procedure using a customizable program. All frames were displayed and automatically analyzed one after the other. If tracking was correct then two digital tags (squares, edge length = 10 pixels ~ 1.28 cm) covered the two color stickers. On rare occasions, the coding software could not accurately localize the colored stickers, e.g., due to uneven light conditions. If the digital tag was not covering the color sticker, the researcher could stop the analysis, rewind to specific frames and manually place the tag onto the sticker. Also, if the subject moved the novel object, the researcher could adjust the software to treat the new position of the object as the center of the arena (see supplementary video “Video_1” in Online Resource 2; also accessible at https://github.com/jinook0707/AMA: “ama_sample_video”).

### Statistics

Three condition-specific variables were created. For novel object, the mean distance (in pixels) of the head tag from the object across all frames per trial was recorded (‘mean dist. to object’). In the case of novel environment: open field, the difference between the minimal and the maximal distance of the head tag from the center of the arena across all frames was calculated per trial; this ‘roaming range’ variable served as an indication for roaming behavior. For novel environment: shelter, the number of frames the animals spent partially (only one tag detected) or fully (no tag detected) in the shelter per trial were summed up and then transformed into seconds to measure ‘shelter usage’. To warrant the planned comparisons between the two species and phases (as outlined in the introduction) and to assess the influence of potentially confounding factors (e.g., testing time, incubation temperature, etc.), the variables “walking distance” and “head movements without walking” (sums of distances in pixels per trial) were investigated in a Generalized Linear Mixed Model (GLMM), as they could be measured in each of the three conditions (number of pixels served as the unit). They were united into a single variable, a ‘movement component’, by conducting a principal component analysis (PCA). The first component explained 95% of the variance (eigenvalue = 1.91, rotation = varimax, see Table 1 for factor loadings) and was extracted after conducting a factor analysis and a Bartlett's test on the correlation matrix (*df* = 1, χ2 = 226.3, *P* < 0.001). This movement component was used as the response variable in the GLMM together with these coefficients: ‘species’ (alligator/caiman), ‘phase’ (1 or 2), ‘condition’ (novel object/novel environment: open field/novel environment: shelter), ‘testing time’ (morning/evening), ‘incubation temperature’ (male/female), and the three two-way interactions between ‘species’, ‘phase’, and ‘condition’. Because the movement components contained negative values, the data were transformed to be positive by adding the absolute value of the most negative data point followed by taking the square root. The GLMM was run using a Gaussian distribution (with a log link function) and contained subject identity as a random effect. The Akaike information criteria (AIC) was used to reduce the full model to find the best fit. Degrees of freedom, the t-distribution, and subsequently the two-tailed p-values were obtained by employing the Kenward–Roger approximation (Halekoh and Højsgaard 2014). During post hoc analysis, pairwise comparisons were conducted using exact Wilcoxon signed-rank tests within species and exact Wilcoxon rank-sum tests (Mann–Whitney *U* tests) between species (Mundry and Fischer 1998). If the animals showed differential changes in behavior between the two phases, delta scores were calculated by subtracting the values from the second phase from those of the first phase for each individual and the scores were used to compare the two species. The *P* values of all pairwise comparisons were checked with sequential Bonferroni-correction (Holm 1979), if the same data was used for more than one comparison. To evaluate individuals’ behavioral consistency over time, the measurements for the two phases per subject were compared using interclass correlation coefficients (ICC). Statistical analysis was performed in R (version 3.0.2 GUI 1.62 for Mac, R packages: lme4, lmerTest, pbkrtest, coin, irr).

**Table 1:** Component matrix of the principal component analysis for the movement component

	PC1
Total walking distance	0.98
Total head movements	0.98
Eigenvalue	1.91
% of variance explained	95

Standardized loadings (pattern matrix) based upon correlation matrix

## Results

### Movement component

**Fig. 2:**
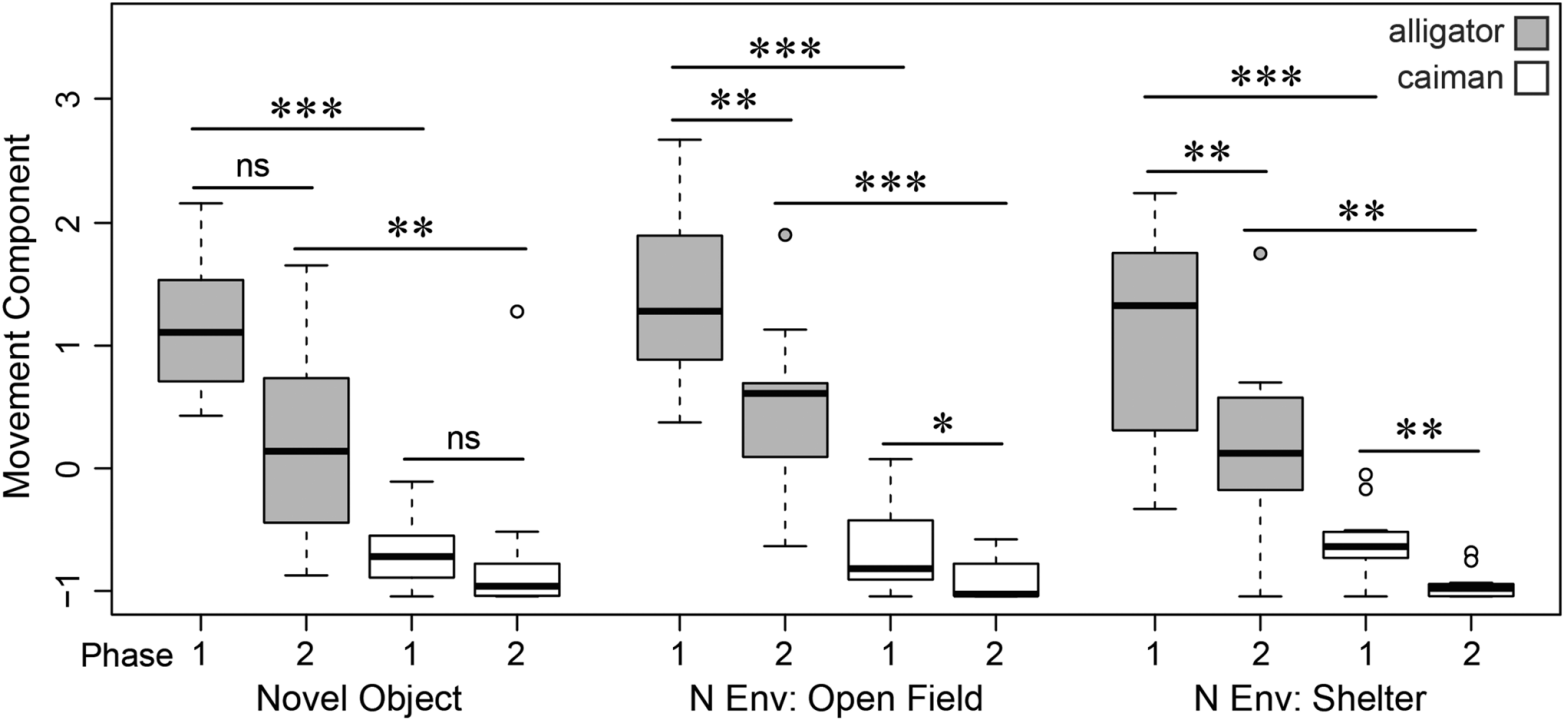
Amount of overall movement by the two species (American “alligator”, spectacled “caiman”) in the three treatments (novel object, novel environment: open field, novel environment: shelter) of the two phases (1–2). Data are represented by the principal component “movement component” comprised of “walking distance” and “head movements without walking” measured in pixels per frame. Box plots represent the 25th and 75th percentiles, the line in the box indicates the median, whiskers represent the non-outlier range and dots are outliers (> *Q*_3_ + 1.5 × *IQR* or < *Q*_1_ − 1.5 × *IQR*). *N Env* novel environment, ***P ≤ 0.001, **P ≤ 0.01, *P ≤ 0.05, *ns* not significant

The final GLMM with ‘movement component’ as response variable included main effects ‘species’, ‘phase’, and ‘testing time’, as well as the interaction between ‘species’ and ‘phase’. ‘Incubation temperature’, and hence presumed sex, did not explain any variance and was not part of the model with the best fit. With the exception of ‘testing time’, all contributing coefficients significantly affected movement behavior (Table 2). Consequently, in the post hoc analyses, the two species and the two phases were compared for each condition-specific measurement. Overall, animals of either species moved less in the second phase of the same condition (Fig. 2). These differences were significant for both species in novel environment: open field and novel environment: shelter (‘movement component’, exact Wilcoxon signed-rank test, *N*_ind/species_ = 11, *Z* ≥ 2.092, *P* ≤ 0.037), but not for the novel object trials (*N*_ind/species_ = 11, *Z* ≤ 1.778, *P* ≥ 0.083). The alligators moved more in each phase of every condition than the caimans (‘movement component’, exact Wilcoxon rank sum test, *N*_ind/species_ = 11, *Z* ≥ -3.090, *P* ≤ 0.002, Table 3).

**Table 2:** Values of the final generalized linear mixed model

Response variable	Coefficients	Estimate	SE	*t*	*P*
Movement component	(Intercept)	1.616	0.036	44.856	< 0.001***
	Species (caiman)	− 0.531	0.046	− 11.467	< 0.001***
	Phase (2)	− 0.188	0.03	− 6.239	< 0.001***
	Testing time (morning)	0.043	0.03	1.44	0.161
	Species (caiman)* phase (2)	0.114	0.043	2.68	0.013*

*SE* standard error

***P ≤ 0.001, *P ≤ 0.05

**Table 3:** Testing matrix for the movement component across species (Alligator/Caiman) and phase (1/2)

	Novel object		N Env: open field		N Env: shelter	
	Alligator 2	Caiman 1	Alligator 2	Caiman 1	Alligator 2	Caiman 1
Alligator 1	*Z* = 1.778 *P* = 0.083	*Z* = − 4.682 *P* < **0.001**	*Z* = 2.845 *P* = **0.002**	*Z* = − 4.682 *P* < **0.001**	*Z* = 2.490 *P* = **0.010**	*Z* = − 4.145 *P* < **0.001**
Caiman 2	*Z* = − 3.090 *P* = **0.002**	*Z* = 0.889 *P* = 0.413	*Z* = − 3.879 *P* < **0.001**	*Z* = 2.092 *P* = **0.037**	*Z* = − 3.287 *P* = **0.001**	*Z* = 2.893 *P* = **0.002**

*N Env* novel environment, *N* = 11

Number behind species name = phase (1 or 2)

In bold font are *P* ≤ 0.05

### Condition-specific variables

#### Novel object

**Fig. 3:**
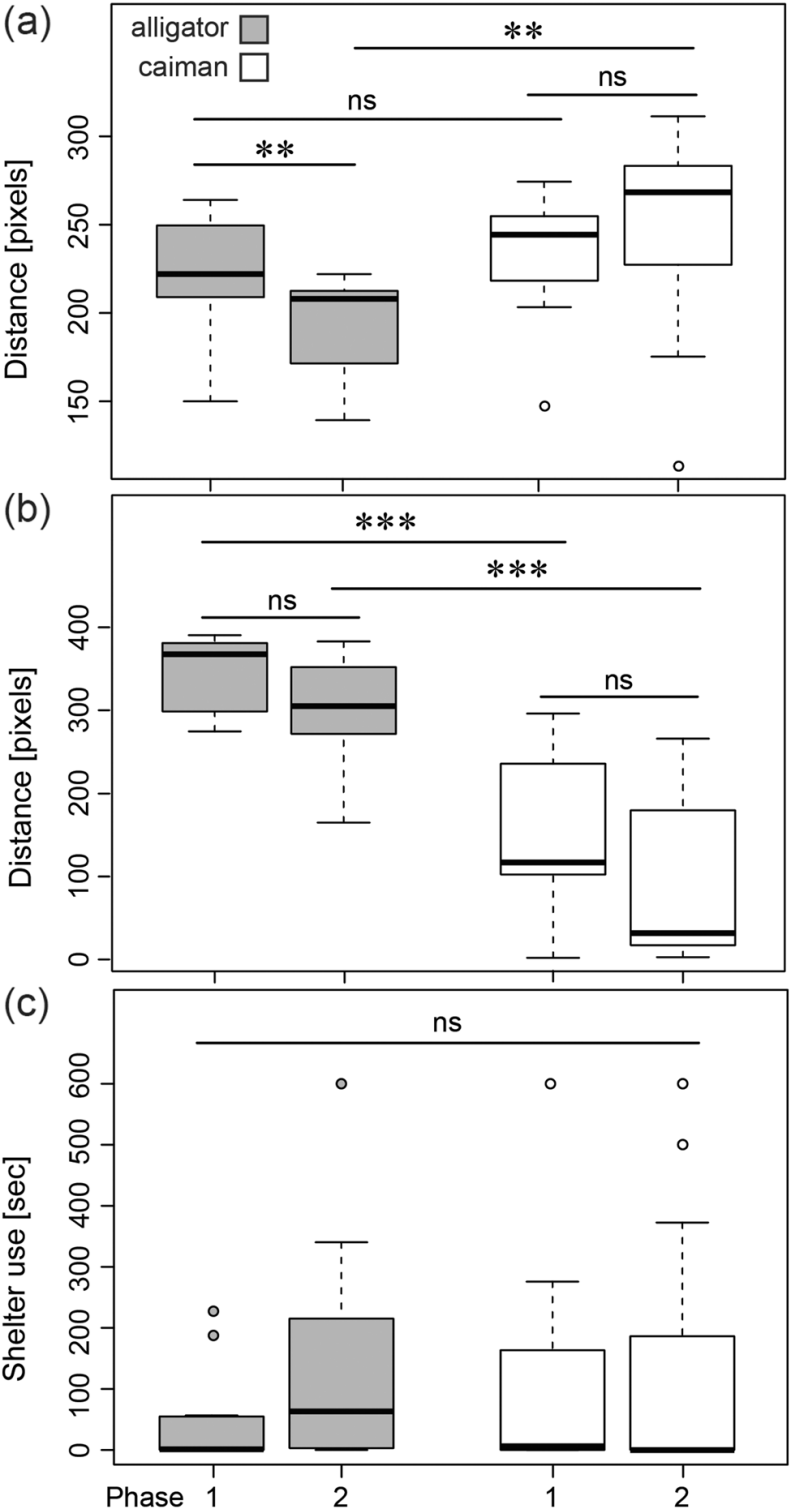
Condition specific variables for the two species (American “alligator”, spectacled “caiman”) in the three conditions **a**–**c** of the two phases. Novel object **a**: the mean distance of the head-tag from the novel object. Novel environment: open field **b**: data represent the roaming range (maximal—minimal distance of head-tag from the arena center). Novel environment: shelter **c**: time spent partially (one tag detected) or fully (no tags detected) in the shelter. Box plots represent the 25th and 75th percentiles, the line in the box indicates the median, whiskers represent the non-outlier range and dots are outliers (> *Q*_3_ + 1.5 × IQR or < *Q*_1_ − 1.5 × *IQR*). ***P ≤ 0.001, **P ≤ 0.01, *ns* not significant

The two species did not differ in mean distance from the object in the first phase (‘mean dist. to object’, exact Wilcoxon rank sum test, *N*_ind/species_ = 11, *Z* = − 0.953, *P* = 0.341), but the alligators significantly reduced that distance in the second phase (exact Wilcoxon signed-rank test, *N*_ind_ = 11, *Z* = 2.491, *P* = 0.008). The caimans showed a non-significant trend to increase the mean distance in the second phase (exact Wilcoxon signed-rank test, *N*_ind_ = 11, *Z* = − 1.868, *P* = 0.064) and the distance clearly differed between the two species in the second phase (exact Wilcoxon rank sum test, *N*_ind/species_ = 11, *Z* = − 2.726, *P* = 0.006, Fig. 3a). A closer examination using delta scores (distance in phase 1 minus distance in phase 2) indicated a diametrical ontogenetic development in exploration behavior in the two species (see Fig. S1, Online Resource 1): nine alligators were on average closer to the novel object in the second phase and nine caimans increased the mean distance to the novel object in the second phase. Overall, the delta scores significantly differed between the two species (novel object-delta scores, exact Wilcoxon rank sum test, *N*_ind/species_ = 11, *Z* = − 2.759, *P* = 0.006).

#### Novel environment: open field

The alligator hatchlings explored a wider area of the arena than the caimans in both phases (‘roaming range’, exact Wilcoxon rank sum test, *N*_ind/species_ = 11, *Z* ≥ − 3.548, *P* < 0.001) but neither species differed between phases (exact Wilcoxon signed-rank test, *N*_ind_ = 11, *Z* ≤ 1.689, *P* ≥ 0.102, Fig. 3b).

#### Novel environment: shelter

Neither species really used the shelter in either phase (Fig. 3c). No differences were observed for ‘shelter usage’ between species (exact Wilcoxon rank sum test, *N*_ind/species_ = 11, *Z* ≤ − 1.228, *P* ≥ 0.219) or phases (exact Wilcoxon signed-rank test, *N*_ind_ = 11, *Z* ≤ − 1.206, *P* ≥ 0.258, see Table 4).

**Table 4:** Testing matrix for the three condition-specific variables across species (Alligator/Caiman) and phase (1/2)

	Mean dist. to object (pixels)		Roaming range (pixels)		Shelter usage (s)	
	Alligator 2	Caiman 1	Alligator 2	Caiman 1	Alligator 2	Caiman 1
Alligator 1	*Z* = 2.491 *P* = **0.008**	*Z* = −0.953 *P* = 0.341	*Z* = 1.334 *P* = 0.206	*Z* = − 3678 *P* < **0.001**	*Z* = − 1.206 *P* = 0.258	*Z* = − 0.572 *P* = 0.567
Caiman 2	*Z* = − 2.726 *P* = **0.006**	*Z* = − 1.868 *P* = 0.064	*Z* = − 3.548 *P* < **0.001**	*Z* = 1.689 *P* = 0.102	*Z* = − 1.228 *P* = 0.219	*Z* = 0.361 *P* = 0.766

*N* = 11; in bold font are *P* ≤ 0.05

### Checking for individual behavioral consistency

Neither species was consistent in its behaviors between the two phases for any of the measurements, with the exception of the caimans showing mediocre consistency in ‘roaming range’ (ICC = 0.489, *F* = 3.39, *P* = 0.035). The alligators showed no consistency in ‘roaming range’ for novel environment: open field (ICC = 0.136, *F* = 1.37, *P* = 0.307). Both, alligators and caimans, were not consistent across the two experimental phases with regards to the two other condition-specific variables, the ‘mean dist. to object’ (alligators: ICC = 0.257, *F* = 2.08, *P* = 0.14/caimans: ICC = 0.212, *F* = 1.51, *P* = 0.259) or ‘shelter usage’ (alligators: ICC = 0.171, *F* = 1.49, *P* = 0.261/caimans: ICC = − 0.199, *F* = 0.806, *P* = 0.63). Neither species showed consistency for the ‘movement component’; this held true for the overall comparison between the two phases (alligators: ICC = 0.2, *F* = 2.01, *P* = 0.102/caimans: ICC = 0.152, *F* = 1.42, *P* = 0.162) and the comparisons between the phases for each of the three conditions, novel object (alligators: ICC = − 0.304, *F* = 0.333, *P* = 0.89/caimans: ICC = -0.216, *F* = 1.5, *P* = 0.266), novel environment: open field (alligators: ICC = 0.412, *F* = 7.39, *P* = 0.121/caimans: ICC = -0.18, *F* = 1.6, *P* = 0.227), and novel environment: shelter (alligators: ICC = 0.432, *F* = 4.26, *P* = 0.075/caimans: ICC = 0.13, *F* = 1.96, *P* = 0.211).

## Discussion

Our findings reveal consistent differences in behavioral predispositions of hatchling American alligators and spectacled caimans across all experimental contexts. The alligators displayed more movement behavior; they covered wider ranges of the novel environments and went closer to novel objects. In contrast, the caimans moved less, covered a smaller proportion of the arena, and stayed further away from the novel objects. The negligible use of the shelter shown by both species indicates that the measured activity across conditions was not motivated by a need to leave the open space; rather suggesting that the alligators indeed showed more exploration behavior than the caimans. In the novel object trials, the alligators consistently decreased the mean distance to the novel object in the second phase, while the caimans even further increased it, indicating that the alligators became more explorative, while the caimans further reduced their activity level. Previous to the experiment, all subjects were exposed to highly comparable surroundings and stimuli in their husbandry, and thus the observed behavioral predispositions are unlikely to be the result of differences in experience. It is in principle possible that American alligator and spectacled caiman juveniles to some extent differ in their husbandry needs, and that the species activity levels were affected by the chosen procedures. However, both species come from comparable habitats and spectacled caimans are invasive in the natural habitat of American alligators. Hence, they were kept under the same conditions after hatching. Interestingly, we found no individual behavioral consistency over the course of the two phases, which further strengthens the hypothesis that the predispositions of American alligators and spectacled caimans are indeed developed during early ontogeny and can be quite different in even closely related crocodilian species.

Crocodilians are an interesting taxonomic order for comparative cognition due to their phylogenetic proximity to birds. The relatively few studies to date taking advantage of this potential usually focused on hatchlings and juveniles (Northcutt and Heath 1971; Sneddon et al. 2000; Somaweera et al. 2011; Vergne et al. 2012) due to the lack of availability of adult subjects and the risks associated with handling them. It is, therefore, important to know whether general conclusions, applicable to the entire order Crocodylia, can be drawn from such studies. We found consistent early-life behavioral differences between two Alligatoridae species. These findings have important implications for comparative cognition. First, even closely related crocodilian species can have substantially varied behavioral predispositions during early ontogeny, and different species might, therefore, not be interchangeable in large scale comparisons. Second, early-life behavioral predispositions appear to be in line with species differences in adult crocodilians, e.g., higher levels of activity in larger species (Grigg and Kirshner 2015), indicating that phylogenetic comparisons can rely on studies focusing on juveniles. The present results add to earlier work, which has shown that crocodilians exhibit species-typical visual signals during social interactions already at the hatchling stage, e.g., raising the head with the snout tip upwards as a sign of submission in several crocodile species (Brien et al. 2013).

As American alligators and spectacled caimans have similar habitats, these early life differences are surprising from an ecological perspective. Both species have a similar range of predators as hatchlings (Somaweera et al. 2013), to which individuals with increased levels of activity could be expected to be more susceptible (Greenberg and Mettke-Hofmann 2001). On the other hand, both species are food generalists (Dodson 1975; Magnusson et al. 1987) and would profit from learning early on about different food sources by means of increased exploration behavior. Differences in innate exploratory tendencies could indicate that two species originate from habitats of different complexity (Mettke-Hofmann et al. 2002). However, the two subject species are native to highly comparable geographical regions (Grigg and Kirshner 2015). Thus, the differences observed are likely due to other factors. In crocodilians, hatchlings are guarded by their mothers (Hunt and Watanabe 1982; Ferguson 1985; Vergne et al. 2009), and in some species by both parents (Lang 1986; Brazaitis and Watanabe 2011), for the first months and up to 3 years after hatching (Trutnau and Sommerland 2006; Thorbjarnarson and Wang 2010; Campos et al. 2012). While an alligator mother can protect her offspring against virtually any natural danger, the protection of a caiman mother might be far from absolute. Thus, it is possible that, as a result of maternal care, alligator hatchlings can afford to be more active and explorative. Future studies investigating this relationship should incorporate additional controls to determine whether parental protection can indeed explain more neophilic tendencies.

Although the effectiveness of parental protection is certainly not the sole aspect influencing the early life behavioral predispositions in crocodilians, it could help explain the success of spectacled caimans as an invasive species. This species has been introduced to the natural habitat of the American alligator, American crocodile, and Cuban crocodile (*Crocodylus rhombifer*), and has established viable populations (Global Invasive Species Database, iucngisd.org: http://www.iucngisd.org/gisd/species.php?sc=1206). All native species are larger than, and probably behaviorally dominant over, the spectacled caiman. Nevertheless, it successfully competes for resources, e.g., small prey items for hatchlings, putting additional pressure on already critically endangered species (Ellis 1980; Powell et al. 2011). If an invasive species such as the spectacled caiman evolved a less active behavioral predisposition due to high predation risk in its natural range, and if such a species were introduced into a habitat, where crocodilians are the apex predators, its hatchlings might have an increased survival rate. It might even be able to outcompete the local crocodilian species, at least in the short term. Over a longer period of time, a species with an innate predisposition for heightened exploration behavior could, however, hold advantages, e.g., because it might explore more potential food sources earlier during ontogeny. Investigating more hatchlings of the same and different species is crucial to gain a full picture of early life behavioral traits in crocodilians.

One limitation of our study is that our subjects were from few broods (two for American alligators, one for spectacled caimans), which makes it possible that a genetic behavioral predisposition was linked to the individual broods rather than the species. This is a general problem for studies investigating young crocodilians; the availability of study subjects of the same age, particularly from more than one species, is usually low (Brien et al. 2013). We are, however, confident that our results indeed reflect behavioral predispositions of the two study species. The incubation conditions differed within the alligators (5 presumably hatched as males, 6 as females) but not in the caimans (11 presumably hatched as males). Incubation conditions, temperature and others, have been demonstrated to significantly impact phenotype and behavior, including the tendency to explore later in life in crocodilians and other non-avian reptiles, independent of kinship (Burger 1991; Sneddon et al. 2000; Deeming 2004; Yowell 2011; Siviter et al. 2017). We controlled for the impact of the two different incubation temperatures and this factor was the first to be excluded during model reduction, indicating that the presumed differences in activity levels originate from an innate predisposition rather than environmental impact. Additionally, the observed plasticity in the novel object trials further suggests that the subjects kept developing the described behavioral traits. Future studies should ideally focus on animals from a larger number of broods and include both sexes for each species.

The discovery that species-typical behavioral predispositions are probably innate in crocodilians provides important implications for conservation efforts intending to release captive-bred individuals into their natural habitat. If juveniles are to be repatriated without the protection of a parent, their initial survival chances could be increased by selecting those with less active behavioral predispositions during early ontogeny. This would particularly be the case for local apex predators (e.g., *Crocodylus siamensis, Crocodylus intermedius*), which might naturally show more exploration behavior than smaller species (e.g., *Crocodylus mindorensis*). It appears evident that a better understanding of crocodilian innate behavior, learning capacities, and ecology will play an important role in supporting conservation and management efforts.

## Supplementary Information

Below is the link to the electronic supplementary material.

Supplementary file1 (PDF 537 KB)

Supplementary file2 (MP4 595 KB)

## Data Availability

The custom software used for the analysis is available under https://github.com/jinook0707/AMA.
