# Supplementary material for: Early life differences in behavioral predispositions in two Alligatoridae species
Source: Anim Cogn. 2021 Jan 17;24(4):753–64. doi: 10.1007/s10071-020-01461-5 (PMC8238711; doi:10.1007/s10071-020-01461-5)
Supplement: Supplementary file 1 — Supplementary file1 (PDF 537 KB) [file 10071_2020_1461_MOESM1_ESM.pdf]

Electronic Supplementary Material

Early life differences in behavioral predispositions in two Alligatoridae species

Stephan A. Reber[1,2,3]*, Jinook Oh[2,4], Judith Janisch[2,5], Colin Stevenson[6], Shaun Foggett[6] & Anna Wilkinson[1,]*

[1]School of Life Sciences, University of Lincoln, Lincoln, UK
[2]Department of Cognitive Biology, University of Vienna, Vienna, Austria
[3]Lund University Cognitive Sciences, Lund University, Lund, Sweden (current address)
[4]Institute of Science and Technology Austria, Klosterneuburg, Austria
[5]Department of Interdisciplinary Life Sciences, University of Veterinary Medicine Vienna, Austria
[6]Crocodiles of the World, Brize Norton, UK

*Correspondence to: mail@stephanreber.com / awilkinson@lincoln.ac.uk

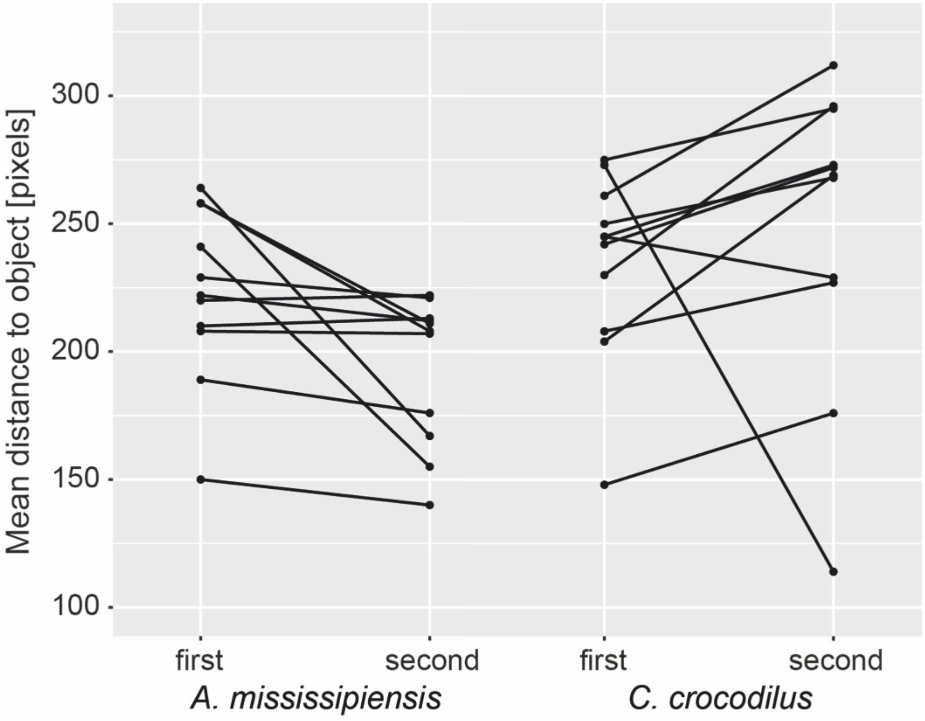

Fig. S1 The contrariwise change of the mean distance (in pixels) to the novel object from the "first" to the "second" phase of the Novel Object condition by the two species, American alligator (*A. mississippiensis*) and spectacled caiman (*C. crocodilus*)

Table S1 Experimental schedule for each individual across the two phases with all conditions

Subject	Age [day]	NO arena	P 1 time	Day 1	Day 2	Day 3	P 2 time	Day 8	Day 9	Day 10
alligator_1	32	black	evening	NE-blue	NO-black-st	Sh-blue+w	morning	NE-blue+c	NO-black_car	Sh-blue+r
alligator_2	32	black	evening	NE-blue+w	NO-black-car	Sh-blue	morning	NE-blue+r	NO-black_st	Sh-blue+c
alligator_3	32	black	evening	NE-blue	NO-black-st	Sh-blue+w	morning	NE-blue+c	NO-black_car	Sh-blue+r
alligator_4	32	blue	evening	NO-blue-car	Sh-black+w	NE-black	morning	NO-blue_st	Sh-black+r	NE-black+c
alligator_5	32	blue	evening	NO-blue-st	Sh-black+w	NE-black	morning	NO-blue_car	Sh-black+r	NE-black+c
alligator_6	26	black	morning	NO-black_car	Sh-blue+r	NE-blue+c	evening	NO-black_st	Sh-blue	NE-blue+w
alligator_7	26	black	morning	NO-black_st	Sh-blue+r	NE-blue+c	evening	NO-black_car	Sh-blue	NE-blue+w
alligator_8	26	blue	morning	Sh-black+r	NE-black+c	NO-blue_car	evening	Sh-black	NE-black+w	NO-blue_st
alligator_9	26	blue	morning	Sh-black+r	NE-black+c	NO-blue_st	evening	Sh-black	NE-black+w	NO-blue_car
alligator_10	26	blue	morning	Sh-black	NE-black+r	NO-blue_car	evening	Sh-black+w	NE-back+c	NO-blue_st
alligator_11	26	blue	morning	Sh-black	NE-black+r	NO-blue_st	evening	Sh-black+w	NE-black+c	NO-blue_car
caiman_1	27	blue	morning	NE-black+w	Sh-black+r	NO-blue_car	evening	NE-black	Sh-black+c	NO-blue_st
caiman_2	27	blue	morning	NE-black	Sh-black+c	NO-blue_st	evening	NE-black+w	Sh-black+r	NO-blue_car
caiman_3	27	blue	morning	NE-black+w	Sh-black+r	NO-blue_car	evening	NE-black	Sh-black+c	NO-blue_st
caiman_4	27	black	morning	Sh-blue+c	NO-black_car	NE-blue+r	evening	Sh-blue	NO-black_st	NE-blue+w
caiman_5	27	black	morning	Sh-blue+c	NO-black_st	NE-blue+r	evening	Sh-blue	NO-black_car	NE-blue+w
caiman_6	28	blue	evening	Sh-black+w	NO-blue_st	NE-black	morning	Sh-black+c	NO-blue_car	NE-black+r
caiman_7	28	blue	evening	Sh-black+w	NO-blue_car	NE-black	morning	Sh-black+c	NO-blue_st	NE-black+r
caiman_8	28	black	evening	NO-black_st	NE-blue+c	Sh-blue+w	morning	NO-black_car	NE-blue+r	Sh-blue
caiman_9	28	black	evening	NO-black_car	NE-blue+c	Sh-blue+w	morning	NO-black_st	NE-blue+r	Sh-blue
caiman_10	28	black	evening	NO-black_st	NE-blue	Sh-blue+r	morning	NO-black_car	NE-blue+w	Sh-blue+c
caiman_11	28	black	evening	NO-black_car	NE-blue	Sh-blue+r	morning	NO-black_st	NE-blue+w	Sh-blue+c

Age=number of days past since hatching on the first day of testing; P=phase; NE=Novel Environment; NO=Novel Object; Sh=Shelter, NO arena=box the subject was habituated to; the car=toy car (blue); st=spinning top (yellow); +w=white wrapping paper; +r=rose wrapping paper; +c=cyan wrapping paper

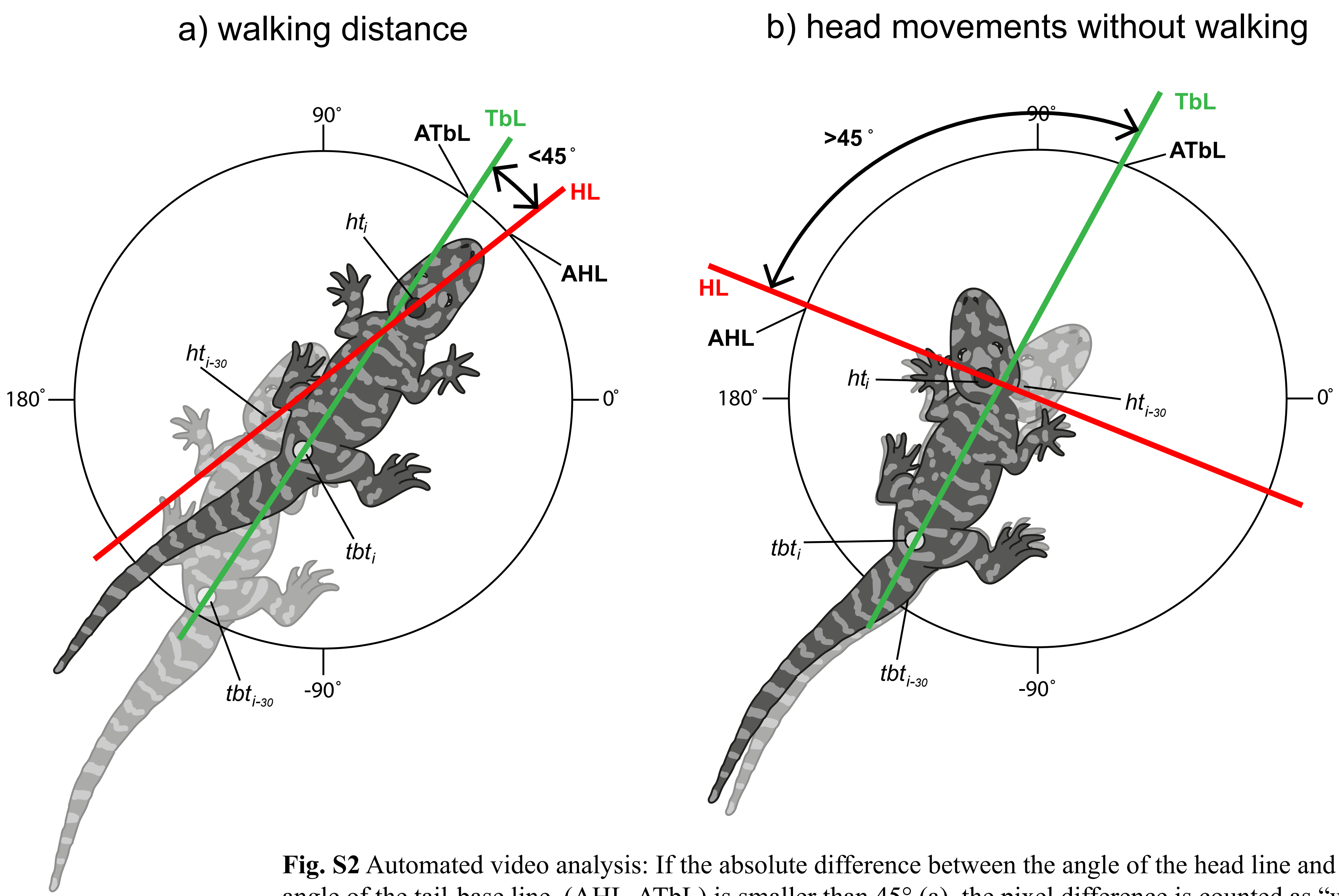

Fig. S2 Automated video analysis: If the absolute difference between the angle of the head line and the angle of the tail-base line (AHL-ATbL) is smaller than 45° (a), the pixel difference is counted as "walking distance"; if it is bigger (b), the pixel margin is recorded as "head movements without walking". HL=head line, TbL=tail-base line, *ht*=head tag, *tbt*=tail-base tag, *i*=current frame, *i-30*=frame 30 frames ago.
